# Cardiac Involvement and Heart Failure Staging in Patients with Systemic Sclerosis Without Pulmonary Arterial Hypertension

**DOI:** 10.3390/jcm14072211

**Published:** 2025-03-24

**Authors:** Maria Isilda Oliveira, Bruno Bragança, José Rodrigues Gomes, Mário Santos

**Affiliations:** 1 Pulmonary Vascular Disease Unit, Department of Cardiology, Unidade Local de Saúde de Santo António, 4099-001 Porto, Portugal; mariossantos001@gmail.com; 2 Department of Immuno-Physiology and Pharmacology, Unidade Multidisciplinar de Investigação Biomédica (UMIB), Instituto de Ciências Biomédicas Abel Salazar (ICBAS), Universidade do Porto, 4050-313 Porto, Portugal; 3 Physical Activity, Health and Leisure Research Centre (CIAFEL), Faculty of Sports, University of Porto, 4200-450 Porto, Portugal; 4 Department of Cardiology, Unidade Local de Saúde Tâmega e Sousa, 4564-007 Penafiel, Portugal; bbraganca14@gmail.com; 5 Department of Immuno-Physiology and Pharmacology, Centro de Investigação Farmacológica e Inovação Medicamentosa (MedInUP), RISE-Health, Instituto de Ciências Biomédicas Abel Salazar (ICBAS), University of Porto, 4050-313 Porto, Portugal; 6 Instituto de Ciências Biomédicas Abel Salazar (ICBAS), University of Porto, 4050-313 Porto, Portugal; 7 ITR—Laboratory for Integrative and Translational Research in Population Health, Rua das Taipas 135, 4050-600 Porto, Portugal

**Keywords:** heart failure, HF staging, systemic sclerosis

## Abstract

**Background/Objectives**: Systemic sclerosis (SSc) is an autoimmune connective tissue disease characterized by fibrosis and vascular damage, significantly increasing the risk of heart failure (HF). **Methods**: This cross-sectional study included 61 SSc patients (92% female, mean age 63 ± 13 years), excluding those with pulmonary arterial hypertension, referred to a tertiary pulmonary hypertension center. HF stages were classified according to updated guidelines. Clinical, echocardiographic, hemodynamic, and functional capacity data were analyzed in relation to HF stages. **Results**: A total of 48% of patients had pre-symptomatic HF (5% stage A, 43% stage B), while 38% had symptomatic HF (stage C). Advanced HF stages were significantly associated with older age (*p* = 0.02) and multiorgan involvement (*p* = 0.045) but not with SSc subtype or autoantibodies. Structural and functional echocardiographic abnormalities were prevalent (77% and 10%, respectively). Markers of elevated ventricular filling pressure such as left atrial volume (*p* = 0.011) and E/e’ ratio (*p* = 0.03) correlated with HF severity. Functional impairment was observed with lower 6 min walk test (6MWT) distance (*p* = 0.017), reduced VO_2_ peak (*p* = 0.015), and increased VE/VCO_2_ slope (*p* = 0.002). Resting pulmonary artery wedge pressure did not correlate with HF stage (*p* = 0.93). VE/VCO_2_ slope and 6MWT were independently associated with HF severity. **Conclusions**: Preclinical and symptomatic HF are highly prevalent in SSc patients. HF staging was linked to disease severity, age, and cardiovascular risk factors. Functional capacity tests (6MWT and CPET) serve as valuable tools for HF risk stratification. These findings highlight the critical need for comprehensive cardiovascular assessment and targeted management strategies to mitigate HF progression in SSc patients.

## 1. Introduction

Systemic sclerosis (SSc) is a rare autoimmune connective tissue disease (CTD) characterized by immune dysregulation, vascular damage, and progressive fibrosis of the skin and other internal organs, resulting in significant morbidity and mortality [1,2]. Based on the extent of skin involvement, SSc is classified as either limited or diffuse [3]. Profiling the expression of specific autoantibodies, including anti-centromere, anti-topoisomerase, and anti-RNA polymerase III, provides important prognostic insights into disease course and outcomes [4]. Pulmonary arterial hypertension (PAH) is one of the most feared cardiovascular complications of SSc and is classically associated with limited SSc and anti-centromere antibodies. However, primary cardiac involvement has been increasingly acknowledged as a common complication of SSc and can be found in both limited and diffuse forms [5]. SSc-related primary cardiac dysfunction results from microvascular dysfunction and inflammatory-fibrotic infiltration, affecting the coronary circulation, conduction system, myocardium, and pericardium [2,5]. Consequently, a broad spectrum of cardiac disorders may develop, including ischemia, arrhythmias, pericarditis, myocarditis, and both ventricular systolic and diastolic dysfunction [6,7].

Due to this diverse cardiac involvement and the common presence of extracardiac manifestations in SSc, diagnosing primary cardiac disease is challenging and requires a high index of suspicion. Most patients present nonspecific symptoms, such as exertional dyspnea and fatigue [8,9]. Diagnosis typically requires a multimodal approach, integrating findings from several techniques, including cardiac imaging, biomarkers, cardiopulmonary function tests, and invasive hemodynamic assessment [2,5]. Several studies have shown that SSc patients have a threefold increased risk of developing heart failure (HF), a disease with a significant impact on their quality of life and survival [6,10]. There is a need for an early diagnosis of primary cardiac cardiomyopathy associated with SSc and the development of specific treatments to prevent the progression of cardiac fibrosis.

HF is currently viewed as a progressive condition that mainly affects people with cardiovascular risk factors (CRFs) (e.g., elderly, hypertension, obesity, diabetes; HF stage A) that will develop several structural and functional cardiac changes over time (left ventricle hypertrophy, diastolic dysfunction, etc.; HF stage B), with the eventual onset of symptoms such as exertional intolerance (HF stage C) and progression to a more severe disease stage with the need for advanced HF therapies (HF stage D). This classification, put forward by the American Heart Association, encourages the diagnosis of HF at an earlier stage and the implementation of treatments that could change its natural history by preventing or postponing its symptomatic stages [11]. Despite a similar prevalence of some common CRFs, SSc patients have a threefold increased risk of experiencing cardiovascular events [12]. Furthermore, left ventricular (LV) diastolic dysfunction is present in one-third of SSc patients and precedes the development of symptomatic HF [13].

In this study, we aimed to assess the prevalence of HF stages in SSc patients without PAH and to study its clinical and functional correlates.

## 2. Materials and Methods

### 2.1. Study Design and Studied Population

We performed an observational cross-sectional study involving consecutive patients referred to the pulmonary hypertension unit at Centro Hospitalar Universitário de Santo António (CHUdSA) since April 2017, who underwent right heart catheterization (RHC) for suspected PAH based on one of the following criteria: an intermediate-to-high echocardiographic probability of pulmonary hypertension (PH), as defined by 2022 ESC/ERS Guidelines for the diagnosis and treatment of pulmonary hypertension [14]; a high probability of PAH according to the DETECT score [15]; or otherwise unexplained exertional dyspnea. Subjects had to be over 18 years of age and had to meet the ACR/EULAR 2013 Classification Criteria for diagnosis of SSc [3]. Clinical data such as age, sex, body mass index (BMI), New York Heart Association (NYHA) functional class, comorbidities, medication, and pulmonary function tests were extracted from clinical records. Patients with a diagnosis of PAH (Group 1 PH) were excluded from this study. The various stages of HF have been defined based on the 2022 AHA/ACC/HFSA Guideline for the Management of Heart Failure [11]. The scope of this study is centered on patients with SSc who are experiencing HF from Stage A to D. Stage 0 indicates no risk factors and no structural heart disease.

### 2.2. Echocardiography

Echocardiographic image acquisition was performed by a certified cardiologist using a Vivid E95 ultrasound scanner with an M5Sc-D Transducer (2–5 MHz; GE Healthcare, Horten, Norway). Standard 2D images with the patient in a left lateral decubitus position were saved in cine loop digital format, as well as static images of pulsed-wave Doppler, continuous-wave Doppler, and tissue Doppler. All images were analyzed offline using EchoPAC software (versions 201 and 206, GE Healthcare, Horten, Norway). Diastolic function was assessed as recommended by current ASE/EACVI guidelines [16], using the following variables: annular e’ velocities (septal and lateral), mitral E velocity, mitral E/e’ ratio (average e’ from septal and lateral walls), left atrial volume index (LAVI), and tricuspid regurgitation peak velocity. For speckle-tracking echocardiography, images (apical four-, two-, and long-axis 2D) were recorded in a digital raw-data format for posterior analysis using EchoPAC software (version 206, GE Healthcare, Horten, Norway), which automatically tracked the myocardial tissue with manual adjustments to region of interest and tracking made when necessary. Global longitudinal strain (GLS) was obtained according to the consensus documents of the EACVI/ASE/Industry TaskForce [17].

### 2.3. Invasive Hemodynamic Assessment

The hemodynamic assessment was performed using a Swan-Ganz catheter by right heart catheterization according to our institution protocol: at rest, with no sedation, through an echo-guided internal jugular vein or brachial vein approach by trained operators. Pulmonary artery, right atrial, and pulmonary arterial wedge pressure (PAWP) were recorded at the end of a quiet respiratory cycle. Cardiac output (CO) was calculated from an average of at least three measurements using the thermodilution method. Pulmonary vascular resistance (PVR) was calculated using the standard formulas [18].

### 2.4. Submaximal and Maximal Exercise Testing

The 6-min walk test (6MWT) was performed in a 30-meter-long corridor under the same environmental conditions. Participants were instructed to walk the maximal distance in 6 min. Resting stops were allowed when patients found it necessary. The equation for reference values used is the one proposed by Enright and colleagues [19]. Blood pressure was measured before and after the test.

All patients able to exercise underwent a maximal cardiopulmonary exercise test (CPET). CPET was performed on a treadmill (Medisoft, Medel 870C) and analyzed through Blue Cherry^®^ Software (version 1.3.3.3, Geratherm^®^ Respiratory GmbH, Bad Kissingen, Germany). CPET parameters were registered every 30 s. The exercise protocol was chosen after a brief interview to assess the patient’s level of physical activity [20]. The patients were encouraged to make maximum effort (peak RER of 1.1).

### 2.5. Statistical Analysis

All data were expressed as mean ± standard deviation (sd) or percentage (%), as appropriate. Histograms were used to assess the normality of data distribution. Patients were divided into groups according to their HF stage. Continuous variables were compared between groups by one-way ANOVA, and categorical variables were compared using the Pearson χ2 test or Fisher exact test, as appropriate. Statistical analyses were conducted using STATA 16.1 (StataCorp LLC, College Station, TX, USA). A two-sided *p*-value < 0.05 was considered significant.

## 3. Results

### 3.1. Population Characteristics

We studied 61 patients with SSc, whose clinical characteristics are presented in Table 1. They were mostly females (92%), with a mean age of 63 ± 13 years. Limited SSc was the most common SSc subtype (75%). Ninety-four percent of the patients presented more than one organ involvement attributed to SSc at the baseline assessment. The skin was the most affected organ (95%), followed by peripheral vessels (80%), the gastrointestinal tract (49%), the lungs (43%), the joints (10%), and the kidneys (1%).

### 3.2. Heart Failure Staging

**Figure 1:**
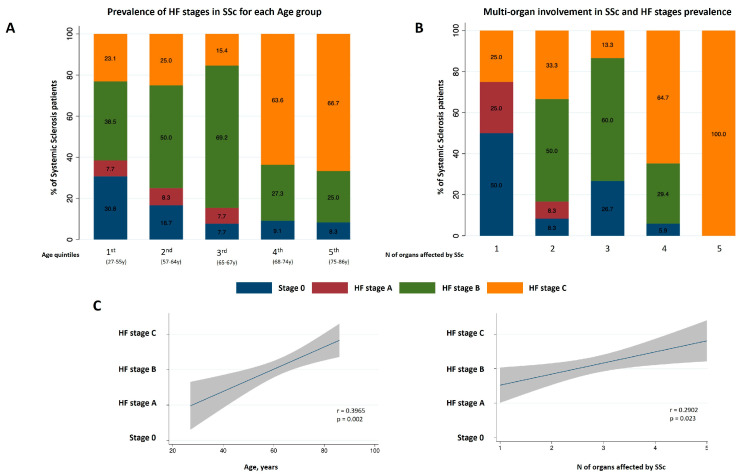
Relationship between heart failure (HF) stages, age and multi-organ involvement in systemic sclerosis (SSc) patients. Panel (**A**), prevalence of HF stages according to age quintiles. Panel (**B**), prevalence of HF stages for the number of organs involved in SSc, excluding cardiac dysfunction. Panel (**C**), linear regression of HF stages with age (left-side plot) and number of organs involved in SSc (right-side plot) with shaded 95% confidence interval. Pearson correlation coefficient (r).

Forty-eight percent of the studied patients were classified into pre-symptomatic HF stages (5% for stage A and 43% for stage B), while 38% had symptomatic HF (stage C). In the HF stage B, 32% of patients showed elevated NT-proBNP levels. No patients were classified into advanced HF stage D. HF stages were significantly correlated with plasmatic NT-proBNP levels and age (Table 1, Figure 1).

Comorbidities not included in HF staging criteria were not associated with HF stages (Table 1). The number of organs affected by SSc, excluding the heart, was associated with HF stages severity (Figure 1). No significant association was found between the organ involved, disease duration, capillaroscopy, SSc antibodies, and HF stages. Regarding treatment at the baseline, neurohormonal blockers (angiotensin-converting enzyme inhibitors, angiotensin II receptor blockers, beta-adrenolytics, and aldosterone receptor antagonists), loop diuretics, statins, and anticoagulants were more frequent in more advanced HF stages. Targeted therapies for SSc, including vasodilators, immunomodulators, and corticosteroids, were similar across HF stages (Table 1).

### 3.3. Cardiac Phenotype and Central Hemodynamics

The index volume of the left atrium (LAVi) differed across HF stages. The ratio between early mitral inflow velocity and mitral annular early diastolic velocity (E/e’), a surrogate of LV filling pressure was also different across HF stages, being higher in those with more advanced HF. Three patients had HF with left ventricular ejection fraction (LVEF) below 50% (LVEF ranging from 46 to 48%). Other echocardiographic parameters, including GLS and LVEF, showed no significant variation among HF stages (Table 2).

In right heart catheterization, three patients with HF stage B presented PH (two of them with group 2 PH and one considered to be multifactorial PH). HF stage C was presented in five patients with group 2 PH. Hemodynamic variables assessed invasively, such as mean pulmonary arterial pressure (MPAP) and PAWP, were equivalent across HF stages (Table 2).

### 3.4. Cardiopulmonary Exercise Test and 6-Minute Walking Test

All patients performed the 6MWT and the walking distance correlated with HF stage severity (Table 3). CPET was performed in 48 patients (79%). At peak, VO_2_, VCO_2_, and PET CO_2_ decreased with HF severity. Conversely, the VE/VCO_2_ slope, a marker of ventilatory inefficiency, increased with HF severity (Table 3).

In multivariate regression analysis, 6MWT distance and VE/VCO_2_ slope were independently correlated with HF stages (Table 4). The results of the regression analysis also showed that while age, dyslipidemia, hypertension, and the number of organs affected by SSc were initially associated with HF stage severity, after adjustment, only hypertension remained statistically significant (Table 4).

## 4. Discussion

The main findings of our study are the following. First, we observed a significant prevalence of symptomatic HF (stage C) in SSc patients referred for suspected, but not confirmed, PAH. Second, 43% of asymptomatic patients were found to be at an increased risk of developing HF (stage B HF). These symptomatic and presymptomatic HF stages were more frequent in elderly patients, in those with cardiovascular risk factors, and in those with multiorgan involvement by the SSc. Third, there were no significant differences in resting invasive hemodynamics, namely PAWP; however, functional parameters such as walked distance at 6MWT, peak VO_2,_ or VE/VCO_2_ slope at CPET correlated with HF staging.

Our findings emphasize the importance of recognizing cardiac involvement in SSc. The prevalence of symptomatic HF (stage C) of 38%, alongside 42% of preclinical HF stage (stage B), suggests a substantial burden of cardiac dysfunction within this population. Symptomatic HF is particularly prevalent in SSc patients with PAH [21,22]. However, evidence on the prevalence of HF in SSc patients without PAH is limited. Jiang et al. found cardiac involvement in 53% of SSc patients, with 4% of them exhibiting symptomatic HF [23]. The higher prevalence of symptomatic HF in our study may be explained by the study design (we studied SSc patients referred for PH suspicion), the older age of our cohort, and the use of the most recent criteria for HF definition [11].

Multiorgan involvement in SSc has been linked to early disease onset and greater disease severity [24,25], although its association with HF is not well documented. Our findings suggest a positive correlation between the extent of multiorgan involvement and HF severity. Therefore, multiorgan involvement in SSc patients should raise clinical suspicion of cardiovascular complications, including HF.

The close association between uncontrolled CRFs and the onset of HF is well established [11]. The prevalence of CRFs in this SSc cohort was notably high. About one-third of patients had hypertension, and that was linked to HF severity. While we did not specifically assess blood pressure control, it is likely that this high-risk population could benefit from lower blood pressure targets (systolic BP < 120 mmHg) to prevent HF, as shown in the SPRINT trial [26]. Diabetes and obesity were also common, affecting 15% and 16% of patients, respectively. These patients may benefit from antidiabetic drugs with favorable effects on weight control and cardiovascular protection, such as GLP-1 receptor agonists and SGLT2 inhibitors [27].

The inflammatory-fibrotic myocardial milieu in SSc is associated with a threefold risk of cardiovascular events compared to the general population [12]. It has been hypothesized that the vascular dysfunction underlying Raynaud’s phenomenon also affects the coronary microvasculature, contributing to ischemic abnormalities detected on cardiac stress imaging despite the absence of epicardial coronary artery disease [5]. This condition, currently defined as ischemia with no obstructive coronary artery disease (INOCA), is frequently observed in SSc patients and is associated with an increased risk of cardiovascular events and mortality [28,29]. Recent advancements in non-invasive cardiac imaging, including coronary flow reserve assessment via coronary computed tomography angiography (CCTA), cardiac magnetic resonance (CMR), and positron emission tomography (PET), may enhance risk stratification and facilitate more tailored therapeutic approaches in this population [30,31].

Most HF patients in stages B and C had preserved LVEF, with a high prevalence of left ventricular diastolic dysfunction (LVDD), which is consistent with prior studies. Reported LVDD prevalence varies widely in the literature (17–67%) depending on the studied population and diagnostic criteria applied [13,21,32]. This supports HFpEF as the predominant type of HF in SSc [10,33]. Therefore, these patients should be treated according to current guideline recommendations, with emphasis on CRF control and the use of SGLT2 inhibitors for prognosis modification [11,34]. Among chronic inflammatory diseases, SSc, along with systemic lupus erythematosus, carries the highest risk of HF development [33]. The prevalence of HFpEF is likely related to chronic low-level systemic inflammation and immune dysregulation, which contribute to myocardial fibrosis and LVDD [35]. Controlling inflammation may prevent HF progression in SSc, though this remains speculative [5,36].

This study also underscores the importance of SSc phenotyping over time, particularly regarding PAH and LVDD development. Pulmonary vasodilators are commonly used to treat patients with PAH associated with SSc (SSc-PAH) and also patients without PAH but with Raynaud phenomena [14]. Therefore, caution should be taken, as, in the presence of LVDD, pulmonary vasodilators can be associated with clinical worsening due to the development of pulmonary edema [37].

Echocardiography and NT-proBNP are important tools to identify patients at risk of HF development among SSc patients. The most common cardiac abnormalities observed were increased left atrial (LA) volume and elevated E/e’ ratio, both of which indicate increased left heart filling pressures—a key feature of HF. Echocardiography is essential as the standard diagnostic tool for characterizing and monitoring heart disease [5]. Annual comprehensive echocardiographic evaluations are recommended for SSc patients to detect early ventricular dysfunction [38]. Echocardiography coupled with GLS technology can be useful in detecting subtle changes in myocardial function in SSc patients. Although we did not find significant differences between clinical and subclinical HF stages, GLS is being increasingly recognized as an important tool for identifying early ventricular dysfunction in asymptomatic SSc patients [39]. However, a major limitation of LVEF, GLS, and other non-invasive echocardiographic markers is their load dependence, which is particularly relevant in SSc. This is due not only to vascular dysfunction and the high prevalence of CRFs but also to the frequent use of vasodilatory therapy, which may complicate the identification of subtle functional abnormalities. Despite being underexplored in this population, myocardial work (MW) assessment by echocardiography represents a promising approach for comprehensive, load-independent myocardial evaluation [40]. Interestingly, MW appears to be significantly reduced in SSc patients despite having a normal echocardiographic assessment on standard evaluation [41]. Complementing myocardial functional assessment, emerging cardiac magnetic resonance (CMR) mapping techniques have shown potential in detecting subtle fibrosis and other pathological changes in SSc [42]. These advances may, in the future, enable the earlier identification of SSc-related cardiomyopathy and subclinical HF stages.

NT-proBNP plays a crucial role in diagnosing HF, and elevated levels should raise suspicion of its presence [11,34]. The DETECT algorithm includes NT-proBNP quantification and is currently recommended for SSc patients [14]. While primarily used for PAH screening, NT-proBNP may also aid in detecting both clinical and subclinical SSc-related cardiomyopathy. However, interpreting elevated NT-proBNP levels requires caution, as various conditions beyond HF can contribute to its increase. Therefore, NT-proBNP should be assessed alongside clinical evaluation and other diagnostic tools, following current recommendations for HF diagnosis and staging [11]. In our study, 32% of stage B patients had elevated NT-proBNP levels. In addition, NT-proBNP has demonstrated diagnostic and prognostic value in asymptomatic SSc patients, with elevated levels being able to predict 3-year mortality [43].

Our data also reveal an association between HF severity and functional impairment in SSc patients without PAH. Lower 6MWT distances were independently associated with HF severity. Although decreased 6MWT performance is expected in symptomatic HF, this study highlights that even in asymptomatic patients, 6MWT may identify individuals at risk of HF progression. Consistent with the link between multiorgan involvement and HF stages, other studies have shown that reduced 6MWT distance correlates with SSc severity [44]. CPET is often used in this population for evaluating dyspnea and assessing the functional impact of SSc. In line with 6MWT, HF severity was associated with reduced peak VO_2_ and ventilatory inefficiency assessed by VE/VCO_2_ slope during CPET [45]. A low VO_2_ peak and a low anaerobic threshold on CPET can differentiate functional impairment due to cardiac involvement from respiratory limitations or pulmonary vasculopathy in SSc [46]. Notably, resting invasive hemodynamic parameters did not distinguish HF stages, emphasizing the diagnostic value of functional tests like 6MWT and CPET in assessing HFpEF and HF staging [47]. Patients in HF stages B and C had a higher proportion of post-capillary hypertension (PAWP > 15 mmHg); however, no differences were found between these groups. Interestingly, Sarma et al. recently questioned the significance of elevated PAWP in limiting exercise capacity in HFpEF patients, suggesting that mechanisms other than PAWP may contribute to dyspnea and exercise intolerance in SSc patients [48].

Our study has some limitations that should be acknowledged. The limited sample size reduces the statistical power to adjust for potential confounders. However, given the rarity of SSc, our sample size is comparable to similar studies. We recruited patients referred to a PH unit, limiting our findings’ generalizability to a more general population of patients with SSc. However, the main conclusions remain valid in this challenging clinical context. The cross-sectional design of our study does not allow us to make temporal inferences between patients classified in the different HF stages. However, it is plausible that the common understanding informed by longitudinal cohorts of at-risk HF patients can be applied to SSc patients. Additionally, a detailed characterization of capillaroscopic patterns and their relationship with cardiac microvascular involvement was not available. Further studies should explore this aspect to enhance our understanding of the interplay between microvascular dysfunction and cardiac involvement in SSc.

## 5. Conclusions

In conclusion, our study reveals a notable prevalence of symptomatic HF in patients with SSc, with 38% meeting the criteria for symptomatic HF (stage C). The observed prevalence of preclinical HF stages underscores the importance of early detection and intervention to prevent the progression to symptomatic HF. Cardiovascular comorbidities are prevalent, and controlling them may significantly impact this high-risk population. Cardiopulmonary exercise capacity was associated with HF severity, suggesting a decline in functional capacity as HF progresses.

## Figures and Tables

**Table 1:** Clinical characteristics of systemic sclerosis patients according to heart failure stages.

	Total	Stage 0	HF Stage A	HF Stage B	HF Stage C	*p*-Value
**N of patients (%)**	61 (100)	9 (15)	3 (5)	26 (43)	23 (38)	
**Age, years (sd)**	62.5 (13.3)	52.9 (17.3)	53.0 (16.8)	62.3 (9.45)	67.7 (12.9)	**0.017**
**Female sex, n (%)**	56 (92)	9 (100)	3(100)	23 (88)	21 (91)	0.690
**Systemic sclerosis (SSc) subtypes, n (%)**						
Limited	46 (75)	6 (67)	2 (67)	20 (77)	18 (78)	0.257
Diffuse	12 (20)	2 (22)	0 (0)	6 (23)	4 (17)	0.240
Sine scleroderma	3 (5)	1 (11)	1 (33)	0 (0)	1 (4)	-
Overlap, n (%) *	13 (22)	5 (56)	0 (0)	6 (23)	2 (9)	**0.029**
**N of organs involved in SSc ^$^ (sd)**	2.8 (1.0)	2.44 (1.0)	1.66 (0.6)	2.73 (0.8)	3.1 (1.1)	**0.045**
**Disease duration of SSc, months (sd)**	75.9 (56.0)	84.4 (55.1)	67.0 (38.2)	66.7 (49.9)	84.7 (65.6)	0.700
**Abnormal capillaroscopy, n (%)**	49 (80.3)	6 (67)	2 (67)	22 (85)	19 (83)	0.720
**Cardiovascular risk factors** ^§^						
Obesity, n (%)	10 (16)	0 (0)	1 (33)	6 (23)	3 (13)	0.330
Hypertension ^φ^, n (%)	19 (31)	0 (0)	1 (33)	5 (19)	13 (57)	**0.005**
Dyslipidemia, n (%)	22 (36)	0 (0)	1 (33)	11 (42)	10 (53)	0.110
Diabetes mellitus ^∫^, n (%)	9 (15)	0 (0)	0 (0)	4 (15)	5 (22)	0.400
Arteriosclerotic disease ^£^, n (%)	2 (3)	0 (0)	0 (0)	0 (0)	2 (9)	0.330
**Echocardiography**						
Structural heart disease ^‡^, n (%)	46 (77)	0 (0)	0 (0)	23 (92)	23 (100)	**<0.001**
Reduced LV/RV function	7 (11)	0 (0)	0 (0)	1 (4)	6 (26)	**0.049**
Ventricular hypertrophy	5 (9)	0 (0)	0 (0)	2 (8)	3 (14)	0.640
Chamber enlargement	38 (62)	0 (0)	0 (0)	18 (69)	20 (86)	**<0.001**
Wall motion abnormalities	-	-	-	-	-	-
Valvular heart disease	5 (8)	0 (0)	0 (0)	1 (4)	4 (17)	0.260
Increased filling pressure ^¥^, n (%)	10 (19)	2 (18)	0 (0)	4 (15)	6 (33)	**0.017**
LAVI > 34 mL/m^2^	33 (55)	1 (12)	0 (0)	14 (54)	18 (78)	**0.002**
Average E/e’ ≥ 15	5 (9)	0 (0)	0 (0)	1 (4)	4 (22)	0.140
Septal e’ < 7 cm/s	18 (33)	0 (0)	0 (0)	8(31)	10 (53)	**0.040**
Lateral e’ < 10 cm/s	31 (56)	0 (0)	1 (33)	16 (62)	14 (74)	**0.006**
TR velocity > 2.8 m/s	14 (24)	0 (0)	0 (0)	6 (23)	8 (36)	0.170
**NYHA functional class, n (%)**						**<0.001**
I	0 (0)	0 (0)	0 (0)	0 (0)	0 (0)	**<0.001**
II	20 (33)	0 (0)	0 (0)	0 (0)	20 (87)	0.407
III	3 (5)	0 (0)	0 (0)	0 (0)	3 (13)	-
**Body mass index, Kg/m^2^ (sd)**	25.8 (5.4)	23.6 (3.0)	28.1 (3.6)	27.5 (6.2)	24.4 (4.9)	**0.010**
**Comorbidities**						
Chronic kidney disease, n (%)	9 (15)	1 (11)	0 (0)	2 (8)	6 (26)	0.280
Atrial fibrillation, n (%)	3 (5)	0 (0)	0 (0)	0 (0)	3 (13)	0.160
Venous thromboembolism, n (%)	2 (3)	0 (0)	0 (0)	0 (0)	2 (19)	0.330
Neoplasia, n (%)	5 (8)	0 (0)	0 (0)	1 (4)	4 (17)	0.230
Asthma, n (%)	2 (3)	0 (0)	0 (0)	2 (8)	0 (0)	0.430
COPD, n (%)	2 (3)	0 (0)	0 (0)	0 (0)	2 (9)	0.330
Obstructive sleep apnea, n (%)	1 (2)	1 (6)	0 (0)	1 (4)	0 (0)	0.710
Interstitial lung disease, n (%)	6 (10)	0 (0)	0 (0)	4 (15)	2 (9)	0.520
**Laboratory values**						
NT-proBNP ≥ 125 pg/mL ^¤^ n (%)	26 (43)	2 (22)	0 (0)	8 (32)	16 (70)	**0.009**
NT-proBNP, pg/mL (sd)	320 (567)	105.7 (73.6)	62.7 (38.4)	160 (228)	612 (810)	**0.015**
Creatinine, mg/dL (sd)	0.74 (0.23)	0.67 (0.10)	0.58 (0.06)	0.72 (0.16)	0.80 (0.32)	0.270
Anti–Scl-70^+^, n (%)	11 (18)	1 (11)	0 (0)	6 (23)	4 (18)	0.710
Anti-RNAP3^+^, n (%)	1 (2)	0 (0)	0 (0)	0 (0)	1 (5)	0.620
Anti-centromere^+^, n (%)	41 (68)	7 (78)	2 (67)	16 (62)	16 (73)	0.770
**Treatment**						
Oxygen therapy, n (%)	2 (3)	0 (0)	0 (0)	1 (4)	1 (4)	0.092
Loop diuretics, n (%)	14 (23)	0 (0)	0 (0)	3 (12)	11 (48)	**0.004**
ACE inhibitors, n (%)	6 (10)	0 (0)	0 (0)	3 (12)	3 (13)	0.650
Beta-adrenolytics, n (%)	7 (11)	0 (0)	0 (0)	1 (4)	6 (26)	0.050
MRA, n (%)	13 (21)	0 (0)	0 (0)	6 (23)	7 (30)	0.220
Calcium channel blockers, n (%)	31 (51)	4 (44)	2 (67)	16 (62)	9 (39)	0.410
Vasodilators, n (%)	12 (20)	4 (44)	0 (0)	5 (19)	3 (13)	0.180
Statins, n (%)	26 (43)	0 (0)	1 (33)	12 (46)	13 (57)	**0.033**
Antidiabetics, n (%)	9 (15)	0 (0)	0 (0)	4 (15)	5 (22)	0.400
Anticoagulation, n (%)	8 (13)	1 (11)	0 (0)	1 (4)	6 (26)	0.120
Primrose oil, n (%)	21 (34)	3 (33)	1 (33)	12 (46)	5 (22)	0.360
Corticosteroids, n (%)	9 (15)	2 (22)	0 (0)	3 (12)	4 (17)	0.740
Immunomodulators, n (%)	15 (25)	1 (11)	0 (0)	8 (31)	6 (26)	0.490

Clinical characteristics of studied patients are presented by HF stages; Stage 0 means absence of any criteria present in HF staging according to 2022 AHA/ACC/HFSA guidelines. * Patients with other connective tissue disease in addition to SSc. ^$^ Number of organs involved in SSc, excluding the heart. ^§^ Coexistence of dyslipidemia and obesity were included as criteria qualifying for HF stage A, being an adaption of 2022 AHA/ACC/HFSA guideline in HF staging as data were not fully available to diagnose metabolic syndrome. ^φ^ Hypertension = systolic blood pressure (BP) > 140 mmHg or diastolic BP > 90 mmHg or antihypertensive pharmacotherapy. ^∫^ Diabetes mellitus = insulin or antidiabetic drugs. ^£^ Arteriosclerotic disease included coronary artery disease, stroke, or peripheral artery disease; however, for this population, documented atherosclerotic disease were restricted to coronary disease. ^¤^ Raised NT-proBNP not otherwise explained (e.g., CKD). The patients included in this cohort also did not have known previous exposure to cardiotoxic agents, genetic cardiomyopathy, or family history of cardiomyopathy. ^‡^ Structural disease included reduced left (LVEF < 50% or GLS < 16%) or right ventricular systolic function (TAPSE < 17 mm or RVFAC < 35%); hypertrophy of either left ventricle (LVMI > 116 g/m^2^ for male and >95 g/m^2^ for female) or right ventricle (RV free wall thickness > 5 mm); RWT > 0.42; wall motion abnormalities at rest were not reported for this cohort; valvular heart disease were considered positive in the presence of at least moderate stenosis or regurgitation in any heart valve. ^¥^ According to the diastolic dysfunction criteria of the current ASE/EACVI guidelines. **Abbreviations:** ACE: angiotensin-converting enzyme; CKD: chronic kidney disease defined as estimated glomerular filtration rate < 60 mL/min/1.73 m^2^; COPD: chronic obstructive pulmonary disease; e’: average peak early myocardial relaxation velocity; E: early mitral valve inflow velocity; RVFAC: Fractional Area Change; GLS: global longitudinal strain; HF: heart failure; LAVi: left atrial volume index; LVEF: left ventricular ejection fraction; MRA: mineralocorticoid receptor antagonist; NT-proBNP: N-terminal pro B-natriuretic peptide; RWT: relative wall thickness; TAPSE: tricuspid annular plane systolic excursion; TR: tricuspid regurgitation; SSc: systemic sclerosis. The bold formatting was used to highlight significant values (*p* < 0.05).

**Table 2:** Echocardiographic, hemodynamic, and pulmonary characteristics in SSc patients.

	Stage 0	HF Stage A	HF Stage B	HF Stage C	*p*-Value
**Echocardiography**					
LVEF, % (sd)	58.3 (4.4)	60.7 (1.5)	62.5 (6.3)	61.7 (8.3)	0.540
GLS, % (sd)	−19.6 (2.3)	−18.5 (-)	−18.8 (2.6)	−18.3 (2.3)	0.830
LAVI, mL/m^2^ (sd)	26.4 (2.5)	28.0 (0.8)	34.3 (9.9)	50.2 (29.4)	**0.011**
LVEDVi, ml/m^2^	43.2 (8.1)	42.6 (10.4)	42.9 (10.2)	45.0 (12.2)	0.940
E/A (sd)	1.41 (0.3)	1.20 (0.58)	1.07 (0.28)	1.06 (0.51)	0.180
E/e’ (sd)	6.85 (1.04)	5.71 (1.32)	9.77 (3.8)	11.6 (5.4)	**0.026**
RVFAC, % (sd)	43.7 (5.4)	51.0 (3.0)	42.6 (6.2)	43.4 (6.7)	0.200
TAPSE, mm (sd)	23.7 (3.1)	20.0 (4.4)	21.5 (2.6)	20.2 (4.8)	0.170
RA, mL/m^2^ (sd)	23.6 (6.9)	20.0 (4.4)	20.8 (5.8)	27.6 (12.8)	0.085
RVEDA, cm^2^/m^2^ (sd)	9.87 (0.9)	10.4 (1.4)	10.1 (1.4)	11.3 (2.4)	0.120
TR, m/s (sd)	2.00 (0.91)	1.63 (1.4)	2.0 (1.3)	2.2 (1.2)	0.820
**Right heart catheterization**					
MPAP, mmHg (sd)	15 (1.41)	18.0 (1.41)	20.6 (5.6)	21.7 (7.7)	0.310
PAWP, mmHg (sd)	9.25 (2.1)	10.5 (3.53)	11.1 (4.9)	11.1 (5.7)	0.930
PAWP > 15 mmHg, n (%)	5 (8.2)	0 (0)	3 (27)	5 (26)	0.560
RAP, mmHg (sd)	4.25 (0.96)	6.5 (3.5)	4.82 (2.6)	5.05 (3.0)	0.820
CI, L/min/m^2^ (sd	3.2 (0.6)	4.25 (0.49)	3.64 (1.14)	3.13 (1.0)	0.340
PVR, WU (sd)	1.12 (0.52)	1.65 (1.5)	1.63 (0.78)	2.33 (1.1)	0.095
**Pulmonary function tests**					
DLCO, % predicted (sd)	79.6 (19.4)	63.5 (-)	70.0 (19.9)	60.8 (14.3)	0.400
FVC, % predicted (sd)	94.1 (8.1)	96.4 (-)	95.4 (24.1)	96.2 (23.4)	1.000
FVC/DLCO, ratio (sd)	1.23 (0.35)	1.51 (-)	1.41 (0.34)	1.65 (0.51)	0.370

**Abbreviations**: CI: cardiac index; DLCO: diffusing capacity of the lungs for carbon monoxide; E/A: ratio of the early to late diastolic transmitral flow velocity; E/e’: average ratio of the early mitral valve inflow velocity (E) to peak early myocardial relaxation velocity (e’); FVC: forced vital capacity; GLS: global longitudinal strain; LAVi: left atrial volume index; LVEF: left ventricular ejection fraction; LVEDVi: left ventricular end-diastolic volume index; MPAP: mean pulmonary arterial pressure; PAWP: pulmonary arterial wedge pressure; PVR: pulmonary vascular resistance (WU, Woods units); RA: right atria; RAP: right atrial pressure; RVEDA: right ventricular end-diastolic area; RVFAC: Fractional Area Change; TAPSE: tricuspid annular plane systolic excursion; TR: tricuspid regurgitation; SSc: systemic sclerosis. The bold formatting was used to highlight significant values (*p* < 0.05).

**Table 3:** Submaximal and maximal functional capacity across HF stages in SSc patients.

	Stage 0	HF Stage A	HF Stage B	HF Stage C	*p*-Value
**Six-minute walk test (6MWT)**					
Distance, m (sd)	508 (109)	414 (33)	378 (114)	360 (91)	**0.017**
% predicted distance (sd)	92.9 (9.5)	84.6 (11.7)	76.9 (21.2)	77.6 (18.5)	0.230
**Cardiopulmonary Exercise Testing**					
Maximal heart rate, % predicted (sd)	92.7 (7.7)	91.3 (10.7)	85.0 (11.7)	79.0 (14.9)	0.057
VO_2_ peak, mL/min/kg (sd)	22.1 (4.8)	17.9 (2.0)	17.1 (4.9)	15.7 (4.3)	**0.015**
% VO_2_ predicted	88.9 (17.1)	84.0 (13.5)	77.9 (23.5)	74.2 (17.7)	0.370
Pulse O_2_ peak (sd)	8.84 (1.5)	7.1 (2.5)	8.94 (2.9)	7.78 (1.5)	0.360
PET CO_2_ peak (sd)	33.8 (3.0)	28.0 (3.6)	33.8 (4.1)	27.9 (4.8)	**0.024**
VE/VCO_2_ slope (sd)	31.1 (3.0)	38.7 (5.0)	32.4 (5.1)	38.9 (7.5)	**0.002**
RER peak, (sd)	1.08 (0.74)	1.02 (0.04)	1.05 (0.13)	1.05 (0.13)	0.880

**Abbreviations:** PET CO_2_: end-tidal carbon dioxide partial pressure; RER: respiratory exchange ratio of carbon dioxide production to oxygen consumption; SSc: systemic sclerosis; VO_2_: O_2_ consumption; VCO_2_: carbon dioxide production; VE: pulmonary ventilation. The bold formatting was used to highlight significant values (*p* < 0.05).

**Table 4:** Univariate and multivariate linear regression models for HF stages in SSc patients.

Variable	Univariate		Multivariate	
	Coefficient (SD)	*p*-Value	Coefficient (SD)	*p*-Value
Age	0.030 (0.009)	**0.002**	0.020 (0.011)	0.062
Sex	−0.40 (0.480)	0.403	0.251 (0.504)	0.621
Hypertension	0.870 (0.260)	**0.001**	0.616 (0.302)	**0.047**
Dyslipidemia	0.589 (0.262)	**0.029**	0.179 (0.300)	0.554
Body mass index	0.001 (0.024)	0.979	0.001 (0.023)	0.959
Diabetes	0.613 (0.361)	0.095	0.291 (0.360)	0.420
Chronic kidney disease	0.484 (0.368)	0.194	0.009 (0.374)	0.980
N organs involved by SSc	0.299 (0.128)	**0.023**	0.258 (0.129)	0.051
6MWT distance	−0.004 (0.001)	**0.003**	−0.003 (0.001)	**0.029**
VO_2_ peak	−0.094 (0.028)	**0.002**	−0.070 (0.036)	0.060
VCO_2_ peak	−0.072 (0.231)	**0.003**	−0.058 (0.030)	0.061
VE/VCO_2_ basal	0.031 (0.019)	0.111	0.038 (0.019)	0.050
VE/VCO_2_ peak	0.060 (0.021)	**0.006**	0.069 (0.022)	**0.003**
VE/VCO_2_ slope	0.060 (0.225)	**0.011**	0.057 (0.023)	**0.018**
PET CO_2_ basal	−0.035 (0.041)	0.384	−0.076 (0.047)	0.110
PET CO_2_ peak	−0.046 (0.030)	0.135	−0.060 (0.031)	0.059

Multivariate model adjusted for age, sex, hypertension, dyslipidemia, diabetes, body mass index, and chronic kidney disease. **Abbreviations**: 6MWT: 6-min walk test; PET CO_2_: end-tidal carbon dioxide partial pressure; SSc: systemic sclerosis; VO_2_: O_2_ consumption; VCO_2_: carbon dioxide production; VE: pulmonary ventilation. The bold formatting was used to highlight significant values (*p* < 0.05).

## Data Availability

On request, the corresponding author must provide access to the database.
